# Diurnal Patterns and Correlates of Older Adults’ Sedentary Behavior

**DOI:** 10.1371/journal.pone.0133175

**Published:** 2015-08-05

**Authors:** Jelle Van Cauwenberg, Veerle Van Holle, Ilse De Bourdeaudhuij, Neville Owen, Benedicte Deforche

**Affiliations:** 1 Department of Public Health, Faculty of Medicine and Health Sciences, Ghent University, Ghent, Belgium; 2 Department of Human Biometry and Biomechanics, Faculty of Physical Education and Physical Therapy, Vrije Universiteit Brussel, Brussels, Belgium; 3 Fund for Scientific Research Flanders (FWO), Brussels, Belgium; 4 Department of Movement and Sport Sciences, Faculty of Medicine and Health Sciences, Ghent University, Ghent, Belgium; 5 Baker IDI Heart and Diabetes Institute, The University of Queensland, Melbourne University and Monash University, Melbourne, Australia; Vanderbilt University, UNITED STATES

## Abstract

**Introduction:**

Insights into the diurnal patterns of sedentary behavior and the identification of subgroups that are at increased risk for engaging in high levels of sedentary behavior are needed to inform potential interventions for reducing older adults’ sedentary time. Therefore, we examined the diurnal patterns and sociodemographic correlates of older adults’ sedentary behavior(s).

**Methods:**

Stratified cluster sampling was used to recruit 508 non-institutionalized Belgian older adults (≥ 65 years). Morning, afternoon, evening and total sedentary time was assessed objectively using accelerometers. Specific sedentary behaviors, total sitting time and sociodemographic attributes were assessed using an interviewer-administered questionnaire.

**Results:**

Participants self-reported a median of 475 (Q1-Q3 = 383–599) minutes/day of total sitting time and they accumulated a mean of 580 ± 98 minutes/day of accelerometer-derived sedentary time. Sedentary time was lowest during the morning and highest during the evening. Older participants were as sedentary as younger participants during the evening, but they were more sedentary during daytime. Compared to married participants, widowers were more sedentary during daytime. Younger participants (< 75 years), men and the higher educated were more likely to engage in (high levels of) sitting while driving a car and using the computer. Those with tertiary education viewed 29% and 22% minutes/day less television compared to those with primary or secondary education, respectively. Older participants accumulated 35 sedentary minutes/day more than did younger participants and men accumulated 32 sedentary minutes/day more than did women.

**Conclusion:**

These findings highlight diurnal variations and potential opportunities to tailor approaches to reducing sedentary time for subgroups of the older adult population.

## Introduction

The health benefits of regular moderate-to-vigorous intensity physical activity for older adults are well-established [1]. Recently, sedentary behaviors, defined as involving prolonged sitting and low levels of energy expenditure [2,3], have been found to be associated with type 2 diabetes, cardiovascular morbidity and premature mortality, independent to the influence of moderate-to-vigorous physical activity [4,5,6,7]. In older adults–the most sedentary age group [8,9]–high levels of sedentary time have been linked to decreased functional fitness [10], increased risk of invasive breast cancer [11], increased metabolic risk, overweight and obesity [12,13,14,15], and higher mortality rates [16,17]. Sedentary time has been reported to reach 10h/day among older adults in the USA [8] and has been found to account for some 75% of Icelandic older adults’ waking time [18]. In order to develop a taxonomy of sedentary behaviors, Chastin et al. [19] conducted a Delphi expert consensus process. The resulting taxonomy proposes that sedentary behaviors can be seen as complex and multi-facetted behaviors, which can be differentiated by their purpose, environment, type, posture, social nature, timing, state of the individual, associated behaviors and measurement. As described in this taxonomy, engagement in sedentary behavior may vary throughout the day [19]. Insights into the diurnal patterns of older adults’ sedentary behavior can help to inform the development of interventions, through identifying those segments of the day (morning, afternoon or evening, for example) that may be targeted to reduce older adults’ sedentary time most effectively. Such a diurnal pattern has been observed for older adults’ physical activity, with older adults being especially inactive during the evening [18,20,21]. While less is known about the diurnal patterns of older adults’ sedentary behaviors, an intervention trial with a small sample of Australian older adults did identify lower volumes of accelerometer-measured sedentary time during the morning, versus the afternoon and evening time [22].

Similar to physical activity, overall sedentary time can encompass several particular behaviors (television viewing, driving, working at a computer, etc.). The multi-facetted nature of sedentary behavior [19] implies that reducing particular sedentary behaviors will require different intervention strategies [23] and different sedentary behaviors might have different health outcomes [24,25]. For example, among Asian adults [25], television viewing time but not computer and reading time was found to be adversely associated with several cardio-metabolic biomarkers. Furthermore, despite the health risks of excessive sitting, some particular sedentary behaviors might entail some benefits for older adults (e.g. computer use to maintain cognitive functioning or car driving to remain mobile) [24,26,27]. This suggests the need to not only examine overall sedentary time but also particular sedentary behaviours among older adults. While devices such as accelerometers can provide an objective measure of older adults’ overall sedentary time, self-report methods are needed to assess engagement in particular sedentary behaviors [8].

Now that the health risks of sedentary behaviors have become apparent, a next phase in the public health research strategy is the identification of subgroups at increased risk for engaging in high levels of particular sedentary behaviors [28]. Furthermore, examining differences in diurnal patterns, total sedentary time and particular sedentary behaviors between subgroups (based on age, gender, marital status, and educational level) can identify subgroups that are most in need for interventions aiming to reduce the particular subsets of sedentary behavior [23].

We examined diurnal patterns of accelerometer-derived measurements, to identify morning, afternoon, and evening levels of sedentary time and whether diurnal patterns differed between older adult subgroups (based on age, gender, marital status, and educational level). We also examined sociodemographic correlates of older adults’ sedentary time and particular sedentary behaviors.

## Methods

### Procedures

Data were collected as part of the Belgian Environment and Physical Activity Study–Seniors (BEPAS-Seniors), which has been described elsewhere [29]. Briefly, participants were recruited and visited at home within 20 neighborhoods in Ghent (Belgium). Ghent is a medium-sized city in Flanders (the Northern, Dutch-speaking part of Belgium). Belgium has a Human Development Index (HDI) of 0.881 (HDI rank = 21) [30]. Ghent consists of 14 boroughs and had 247,941 inhabitants and a residential density of 1,570 inhabitants/km^2^ in 2012. Ghent hosts several tertiary education institutions with a total of 68,039 students. The old age dependency ratio (number of older adults / number of 20-to-64-year-olds) and the unemployment rate were 27 and 7%, respectively [31,32].

The 20 neighborhoods were selected from the 201 existing statistical sectors in Ghent. A statistical sector includes about 1,000 inhabitants and it is the smallest unit for which sociodemographic information is available in Belgium. Each neighborhood contained one to five adjacent statistical sectors. Neighborhood selection was based on maximizing the variability in neighborhood environmental characteristics (i.e., neighborhood walkability) and mean annual household income per neighborhood. Stratified sampling based on gender and age (65–74 vs. ≥ 75 years) was used to recruit participants within each neighborhood. The legal pension age in Belgium is 65 years, the average age at retirement is 59.5 years [33] and we visited the participants at home during daytime, therefore, it can be assumed that almost all of the participants were retired. For inclusion, participants had to be non-institutionalized and not limited by their health to walk a couple of 100 meters. This resulted in 508 participating, yielding a response rate of 44.8% (508/1,135 eligible participants found at home). Data were collected by trained researchers between September 2010 and October 2012. The study protocol was approved by the Ghent University Hospital.

During a first home visit written informed consent was obtained and a structured interview assessed health status, physical activity and sedentary behaviors. The participant was also provided with an accelerometer to wear during the next seven days and an appointment for a second home visit approximately eight days later was made. During this second home visit a structured interview assessed sociodemographics, weight and height were measured and the accelerometer was collected.

### Measures

#### Sociodemographics and health status

The following sociodemographic factors were assessed: age, gender, marital status, educational level, and (former) occupation. Age was dichotomized into 65–74 years and ≥ 75 years. Participants were asked for their current marital status: married, widowed, divorced, never been married or cohabiting. Based on exploratory analyses the categories of married and cohabiting and the categories divorced and never been married were collapsed. Educational level was assessed as follows: “What is the highest educational degree that you have obtained?”. Six possible answers were provided ranging from primary education to university. These categories were recoded into: primary, secondary, and tertiary education.

The validated SF-36 [34,35] was used to assess self-rated health (excellent, very good, good, fair or poor) and health limitations to walk more than 1 km (severely, somewhat or not limited). To calculate BMI, height and weight were measured with a SECA 214 stadiometer and a SECA 813 Robusta weight scale up to 0.1 cm and 0.1 kg, respectively.

#### Accelerometer-derived sedentary time

Participants were instructed to wear an Actigraph GT3X+ accelerometer for seven consecutive days. Data registration occurred in 1 min epochs. Twenty-eight participants had no accelerometer data due to device-failure. A period of at least 90 minutes of consecutive zeros was defined as non-wear time [36]. A valid day was defined as a day that contained at least 10 hours of accelerometer data and participants with less than five valid days were excluded from further analyses (n = 25) [37,38]. Participants with more than 18 valid hours/day were also excluded (n = 6). Minutes with less than 100 activity counts were defined as sedentary minutes [39,40]. Moderate-to-vigorous physical activity (MVPA) was defined as ≥ 1952 counts per minute [41]. Accelerometer-derived sedentary time and MVPA were expressed in minutes/day. To examine the diurnal pattern of sedentary time, days were divided into three segments: morning (7h–12h), afternoon (12h–17h), and evening (17h–23h). These segments were selected based on an exploratory analysis of the data. Within each segment, average minutes of sedentary time/hour and MVPA/hour were calculated.

#### Self-reported sedentary behaviors

Participants completed an interview-administered sedentary behavior questionnaire including 12 particular sedentary behaviors; TV viewing, computer use, reading, sedentary hobbies (e.g. handicraft, playing cards), talking/listening to music, telephone use, public transport, driving a car, being passenger in a car, sitting during household chores, resting, and eating. This questionnaire first assessed how many days a certain sedentary behavior was performed in the last seven days. Then it prompted how long, on average, the participant engaged in that sedentary behavior on such a day. The average daily time spent in these sedentary behaviors was calculated: (number of days engaged in the behavior * average time engaged in the behavior on such a day) / 7. The average daily times spent in the different sedentary behaviors were summed to create the variable ‘self-reported total sitting time’. Acceptable test-retest reliability (ICC> 0.70) was found for TV viewing, computer use, driving a car, and total sitting time [42]. These variables acted as dependent variables in the current study. Participants with self-reported total sitting times higher than 18h/day (n = 7) were excluded. This resulted in the inclusion of 442 participants with complete questionnaire and accelerometer data with a mean of 15.0 ± 1.4 valid accelerometry hours/valid day. Participants with valid questionnaire and accelerometer data were significantly younger (74.2 ± 6.2 vs. 76.3 ± 6.4 years, t = -2.5, p = 0.01) and had a lower BMI (22.3 ± 3.6 vs. 23.7 ± 3.9 km/m^2^, t = -2.8, p = 0.01) than participants with invalid data. Participants with valid versus invalid questionnaire and accelerometer data did not differ in their gender distribution, marital status, education level and self-rated health (all p’s > 0.05). Correlation analysis between self-reported total sitting time and accelerometer-derived sedentary time yielded a Spearman’s ρ of 0.30 (p< 0.001) [42].

### Statistical analyses

Statistical analyses were performed using R version 3.03 and significance level was defined at 0.05. All analyses were adjusted for accelerometer-derived MVPA. Analyses with accelerometer-derived sedentary time as the dependent variable were also adjusted for number of valid days and average number of valid hours/valid day.

#### Diurnal patterns of accelerometer-derived sedentary time

To identify the diurnal patterns of accelerometer-derived sedentary time, 3-level models were constructed (segment of the day clustered within participants and participants clustered within neighborhoods) using the LMER-function available in the lme4-package (http://cran.r-project.org/web/packages/lme4/index.html). Since the dependent variable was normally distributed, general linear mixed models were fitted using maximum likelihood. First, we ran a basic model that included the main effects of diurnal pattern, adjusted for accelerometer-derived MVPA, number of valid days and average number of valid hours/valid day. Second, we added the four sociodemographic factors to this basic model. Third, we constructed four separate models by adding the interaction effect between diurnal pattern and one of the sociodemographic factors to the model fitted in step 2. Fourth, we combined all significant interaction effects observed in step 3 into the model fitted in step 2. Finally, non-significant interaction effects were deleted from the model. Graphs were created to facilitate interpretation of the interaction effects [43].

#### Sociodemographic correlates of sedentary behaviors

All dependent variables, except for accelerometer-derived sedentary time, were non-normally distributed. Therefore, generalized linear mixed models (GLMMs), adjusting for the clustering of participants within neighborhoods, were fitted with the GLMER-function available in the lme4-package [44]. Appropriate variance and link functions were selected based on Akaike’s Information Criterion (AIC). For accelerometer-derived sedentary time, a general linear mixed model was fitted (i.e. a Gaussian variance and identity link function) using maximum likelihood. A GLMM with gamma variance and log link function was selected for the dependent variables ‘TV viewing’ and ‘total self-reported sitting time’. Since the dependent variables ‘driving a car’ and ‘computer use’ contained an excessive number of zeros, hurdle models were fitted to these variables: within a hurdle model, two separate analyses are performed. First, a logistic regression model was fitted to estimate the relationship with the odds of participation in (any) car driving/computer use. Secondly, a gamma regression model with log link function was fitted to estimate the relationship with the amount of car driving/computer use among those participants that reported any car driving/computer use in the last 7 days. GLMMs were fitted by Adaptive Gauss-Hermite Quadrature with 25 quadrature points [44]. A backward selection procedure based on the AIC was performed to select the included variables. This implies that the final models included the sociodemographic variables that improved the model fit (irrespective whether they were significantly related to the dependent variable) and accelerometer-derived MVPA.

## Results

### Sample characteristics

Table 1 presents the sample’s descriptive characteristics. Participants had a mean age of 74 ± 6 years and 55% were women. Most participants (66%) were married or cohabiting, 38% had followed tertiary education and 55% had performed a white collar job. Mean BMI was 22 ± 4 kg/m^2^, 18% rated their health to be fair or poor and 27% reported to be limited by their health to walk more than one kilometer. Participants reported a median sitting time while driving a car of 9 (Q1-Q3 = 0–23), while using the computer of 0 (Q1-Q3 = 0–60) and while viewing television of 177 (Q1-Q3 = 90–240) minutes/day. Participants self-reported a median of 475 (Q1-Q3 = 383–599) minutes/day of total sitting time and accumulated a mean of 580 ± 98 minutes/day of accelerometer-derived sedentary time. In comparison to the Flemish population of older adults, our sample was highly educated (38 vs. 16% with tertiary education) [45], had a lower BMI (a mean of 22 vs. 26 kg/m^2^) [46] and viewed less television (a mean of 170 vs. 255 minutes/day) [47].

**Table 1:** Descriptive Characteristics of the Sample.

Characteristics	Total sample(n = 442)
Age (M ± SD)	74.2 ± 6.2
Gender (% women)	54.8
Marital status (%)	
Widowed	21.2
Never married / divorced	12.7
Married / cohabiting	66.1
Educational level (%)	
Primary education	25.8
Secondary education	36.1
Tertiary education	38.1
Occupation (%)	
Household	18.0
Blue collar	26.9
White collar	55.1
BMI (M ± SD)	22.3 ± 3.6
Self-rated health (% fair/poor)^a^	18.1
% limited to walk more than 1 km^a^	26.5
Accelerometer-derived MVPA (Med; Q1-Q3)^b^	10.4; 3.4–23.9
Driving a car (Med; Q1-Q3) ^b^	8.6; 0.0–22.9
Computer use (Med; Q1-Q3) ^b^	0.0; 0.0–60.0
TV viewing (Med; Q1-Q3) ^b^	176.7; 90.0–240.0
Total self-reported sitting time (Med; Q1, Q3) ^b^	475.0; 383.0–599.0
Accelerometer-derived sedentary time (M ± SD)^b^	580.4 ± 97.7

M = mean; SD = standard deviation, Med = median, Q1 = quartile 1, Q3 = quartile 3

^a^ Derived from the SF-36 questionnaire

^b^ Expressed in minutes/day

### Diurnal patterns of accelerometer-derived sedentary time

Overall, accelerometer-derived sedentary time was lowest during the morning and highest during the evening. During the morning, participants spend 30.20 (S.E. = 0.34) minutes/hour sedentary. This increased with 9.64 (S.E. = 0.32, p< 0.001) and 10.88 (S.E. = 0.34, p< 0.001) minutes/hour during the afternoon and evening, respectively. The difference of 1.23 (S.E. = 0.34) minutes/hour between afternoon and evening was also statistically significant (p< 0.001).

**Fig 1:**
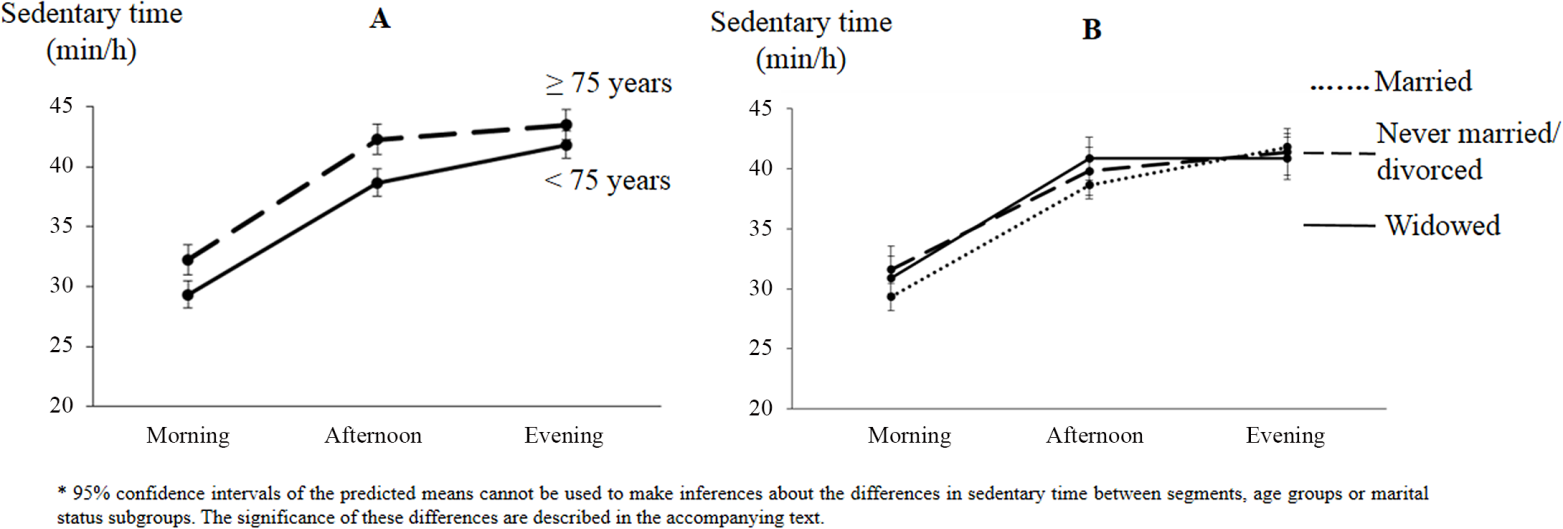
Diurnal patterns of sedentary behavior differed significantly according to age group (panel A) and marital status (panel B).

Significant interaction effects with diurnal pattern were observed for age and marital status (Table 2). In both age groups sedentary times increased throughout the day (Fig 1A), but the increase between afternoon and evening was significantly stronger in participants younger than 75 years compared to those aged 75 years and older (b = 1.93, SE = 0.66, p< 0.01). This resulted in participants younger than 75 years being as sedentary as those aged 75 years and older during the evening, while during the morning (b = 2.90, SE = 0.61, p< 0.001) and afternoon (b = 3.58, SE = 0.62, p< 0.001) the older age group was more sedentary than the younger group.

**Table 2:** Results of the Final Model for the Diurnal Patterns.

	b (S.E.)	p
**Intercept**	28.86 (0.64)	
**MAIN EFFECTS**		
**SEGMENT OF THE DAY** (ref. = morning)		
Afternoon	9.32 (0.45)	< 0.001
Evening	12.47 (0.47)	< 0.001
**Age** (ref. = < 75 years)	2.90 (0.61)	< 0.001
**Gender** (ref. = men)	-1.86 (0.47)	< 0.001
**Education** (ref. = primary)		
Secondary	0.48 (0.58)	0.42
Tertiary	0.52 (0.60)	0.38
**Marital status** (ref. = married/cohabiting)		
Widowed	1.60 (0.76)	0.04
Never married / divorced	2.26 (0.90)	0.01
**INTERACTION EFFECTS**		
Pattern * Age		
Afternoon * ≥ 75 years	0.68 (0.65)	0.30
Evening * ≥ 75 years	-1.24 (0.65)	0.06
Pattern * Marital status		
Afternoon * widowed	0.59 (0.81)	0.47
Evening * widowed	-2.54 (0.81)	<0.01
Afternoon * Never married / divorced	-1.12 (0.96)	0.25
Evening * Never married / divorced	-2.66 (0.96)	<0.01

This model was adjusted for accelerometer-derived MVPA, number of valid days and number of valid hours/valid day (centered around their grand mean).

Sedentary time significantly increased between morning and afternoon in the three marital status subgroups (Fig 1B). Between afternoon and evening sedentary time significantly increased among married participants (b = 3.15, SE = 0.48, p< 0.001), but not among non-married (b = 1.60, SE = 0.95, p = 0.09) and widowed participants (b = 0.01, SE = 0.83, p = 0.98). During the morning, widowed (b = 1.60, SE = 0.76, p = 0.04) and non-married participants (b = 2.26, SE = 0.90, p = 0.01) were more sedentary compared to married participants. The difference between widowed and non-married participants was not statistically significant (b = 0.67, SE = 1.04, p = 0.52). During the afternoon, widowed participants were more sedentary compared to married participants (b = 2.18, SE = 0.76, p< 0.01), but no other significant differences between marital status subgroups were observed. During the evening, no statistically significant differences in sedentary time were observed between the three marital status subgroups.

### Sociodemographic correlates of sedentary behaviors

Results of the final models are presented in Table 3. In the logistic model for driving a car, younger participants and men were, respectively, 2.38 (1 / 0.42) and 5.00 (1 / 0.20) times more likely to have driven a car. Participants with secondary or tertiary education were respectively 2.01 and 2.73 times more likely to have driven a car in the last seven days compared to those with primary education. In the gamma model we observed that, among those who had driven a car in the last 7 days, older participants and women drove 24% minutes less compared to younger participants and men.

**Table 3:** Results of the Final Models for the Relationships between Socio-demographic Factors and Sedentary Behavior(s).

	Driving a car		Computer use		TV viewing	Accelerometer-derived sedentary time^4^	
	Logistic model^1^	Gamma model^2^ (n = 272)	Logistic model^1^	Gamma model^2^ (n = 218)	Gamma model	Gaussian model	
	OR (95%C.I.)	Exp b (95% C.I.)^3^	OR (95%C.I.)	Exp b (95% C.I.) ^3^	Exp b (95% C.I.)^3^	b (S.E.)	p
**Age** (ref. = < 75 years)	0.42 (0.26, 0.68)***	0.76 (0.59, 0.99)*	0.32 (0.20, 0.50)***			35.11 (7.42)	<0.001
**Gender**(ref. = men)	0.20 (0.12, 0.32)***	0.76 (0.59, 0.97)*	0.59 (0.38, 0.91)*	0.59 (0.45, 0.78)***		-32.26 (7.07)	<0.001
**Education**(ref. = primary)							
**Secondary**	2.01 (1.12, 3.60)* ^a^		3.35 (1.86, 6.01)*** ^a^	1.26 (0.79, 2.02) ^a^	0.91 (0.29, 1.16)^a^	12.08 (9.22)^a^	0.19
**Tertiary**	2.73 (1.46, 5.12) ** ^a^		8.54 (4.66, 15.66)*** ^b^	1.63 (1.03, 2.59) * ^a^	0.71 (0.55, 0.90)** ^b^	18.23 (9.75)^a^	0.06

*p< 0.05

**p< 0.01

***p< 0.001

OR = odds ratio, C.I. = confidence interval, S.E. = Standard Error

^a,b^ Categories with different indices differ significantly (p< 0.05). Each column represents the results of one statistical model. All models were adjusted for accelerometer-derived moderate-to-vigorous physical activity (centered around its grand mean).

^1^ The logistic model estimates the relationships between the independent variables and the odds of having driven a car / used the computer in the last 7 day.

^2^ The gamma model estimates the relationships between the independent variables and the amount of driving a car / computer use (in minutes/day) among those who have driven a car / used the computer in the last 7 days.

^3^ Exp b = exponent of b, all gamma models were fitted using a log link function, the exponent of the b’s can be interpreted as a proportional increase in the dependent variable (in minutes/day) with a one-unit increase in the independent variable.

^4^ Adjusted for accelerometer-derived moderate-to-vigorous physical activity, number of valid days and number of valid hours/valid day (centered around their grand mean). The intercept for this model was 603.30 (S.E. = 13.85) minutes/day.

In the logistic model for computer use, younger participants and men were 3.13 (1 / 0.32) and 1.69 (1 / 0.59) times more likely to have used the computer compared to older participants and women, respectively. Participants with secondary and tertiary education were, respectively, 3.35 and 8.54 times more likely to have used the computer in the last seven days compared to participants with primary education. Compared to those with only secondary education, those with tertiary education were 2.55 (95% C.I.: 1.55, 4.18) times more likely to have used the computer. In the gamma model, among those who had used the computer, women reported 41% minutes of computer use less than men. Participants with tertiary education reported 63% more minutes/day of computer use compared to those with primary education. There was no significant difference in volume of computer use between those with primary and secondary and between those with secondary and tertiary education.

For television viewing, there was no significant difference between those with primary and secondary education, but participants with tertiary education viewed 29% and 22% (95% C.I.: 3–37%) minutes/day less television compared to those with only primary or secondary education, respectively.

A significant positive relationship was observed between age and accelerometer-derived sedentary time: older participants accumulated 35.11 sedentary minutes/day more compared to their younger counterparts. Women accumulated 32.26 sedentary minutes/day less compared to men.

No significant relationships between sociodemographic factors and self-reported total sitting time were observed. No significant relationships were observed for marital status.

## Discussion

We identified diurnal patterns that differed between sociodemographic subgroups and sociodemographic correlates of overall sedentary time and particular sedentary behaviors. Our finding that older adults were especially sedentary during the evening, confirms findings from a study among Australian older adults [22]. Furthermore, it is in line with previous studies reporting lower accelerometer counts during evening hours [18,48]. We observed interaction effects with diurnal patterns for age and marital status. These interaction effects indicated that the older age group and widowed participants were more sedentary during the morning and afternoon compared to the younger and non-widowed subgroups, but that there were no differences during the evening. For intervention development, this suggests targeting reductions in sedentary behavior during the evening as a general goal for older adults. The most effective strategies might focus upon reducing or breaking up television viewing, a typical evening activity and responsible for about 35% of the overall self-reported sitting time in our sample. Interventions targeting reductions in daytime sedentary behavior should devote special attention to older adults aged ≥ 75 years or widowed.

Our finding that participants aged 75 years and older accumulated more accelerometer-derived sedentary time than those aged 65 to 74 years confirms previous findings [49,50]. By contrast, we found that the older age group reported to be less likely to have driven a car and to drive less if they had driven a car. Furthermore, the older age group was less likely to have been sitting while using a computer. Possibly, the older age group engages more frequently in other particular sedentary behaviors (e.g. resting, sedentary hobbies) for which we did not study the correlates. Women reported to be less likely to have been sitting while driving a car and while using the computer and reported less minutes when they did engage in these sedentary behaviors. Furthermore, women accumulated approximately 30 minutes/day of accelerometer-derived sedentary time less than men. This supports previous studies reporting older women to be less sedentary than older men [18,49,50].

Those with secondary and tertiary education had higher odds of sitting while driving a car. This might be explained by higher educated participants having higher incomes, which has been linked to higher levels of car and driving license ownership [51]. Those with a higher education were also more likely to have been sitting while using the computer. Furthermore, among those who had used the computer, those with tertiary education had higher volumes of computer use than those with primary education. Higher levels of computer use in older adults with higher education is possibly explained by earlier occupational computer use. In older adults with lower education, the lower computer use might have been compensated for by higher television viewing levels, resulting in no differences in self-reported total sitting time and accelerometer-derived sedentary time according to educational level. This illustrates the need for differentiating between different types of sedentary behaviors. While being as sedentary overall compared to their more-highly educated counterparts, older adults with lower educational attainment might be at increased risk for certain health outcomes due to their higher levels of TV viewing time. Previous studies have linked higher levels of television viewing to higher metabolic risk, decreased grip strength and cognitive functioning, whereas higher levels of computer use were related to increased grip strength and cognitive functioning [24,25,52]. Based on these and current findings, priority should be given to interventions targeting reductions in television viewing time among older adults with lower education.

In the behavioral epidemiology framework described by Sallis and colleagues [28], studying the factors that influence health behaviors (such as sedentary behavior) is considered the third research phase after establishing the links between the behavior and health outcomes and the development of measurement methods. Within this third phase, the identification of sociodemographic correlates of sedentary behavior is a primary purpose. Such a descriptive approach is necessary to identify subgroups of the population who are most in need for interventions. However, as proposed in the seminal work of Rose [53], in order to benefit population health more broadly, a second purpose should be to identify the environmental and other causes of high levels of sedentary time. Socio-ecological models propose individual, physical and social environmental, policy and economic factors that may influence sedentary behavior(s) and can guide such research [23,54].

We identified variations in diurnal patterns and sociodemographic correlates of sedentary behavior(s) which provides basic, but necessary, insights into the emerging field of older adults’ sedentary behavior. This information can inform future intervention development. Furthermore, in line with the multi-facetted nature of sedentary behavior [19] and the differential health outcomes [24,25], we investigated the sociodemographic correlates of total sitting time/accelerometer-derived sedentary time and specific types of sedentary behaviors. However, a limitation of our study is the use of accelerometers to assess overall sedentary time as they cannot distinguish between different postures. However, accelerometers have been shown to have minimal bias compared to the activPAL-device (which does capture body position) [8]. Secondly, our self-report measure of total sitting correlated relatively weakly to accelerometer-derived sedentary time (ρ = 0.30) [42] and we found both measures to relate differently to sociodemographic characteristics. Therefore, it would be helpful to include both self-report and objective measures of sedentary behavior in future studies. Thirdly, our sample was highly educated, viewed more television and had a higher BMI compared to the general population of Flemish older adults. We showed that levels of car driving, computer use and television viewing differed between older adults with different educational backgrounds. Therefore, the observed levels of engagement in sedentary behaviors should not be generalized to the Flemish population of older adults. However, the observed diurnal patterns did not differ according to educational attainment, suggesting that the observed patterns (increasing sedentary time throughout the day) may be applicable to older adults with a high as well as a low educational level. We did not examine the relationships between BMI and (patterns of) sedentary behaviors. Previous studies among older adults have reported higher levels of BMI to be related to higher levels of sedentary behaviors [12,13,15]. Future research could examine whether the diurnal patterns of older adults’ sedentary behaviors differ according to BMI. Fourthly, we were limited to study only three particular sedentary behaviors, because the questions targeting the remaining behaviors were not found to be reliable [42]. Fifthly, while we investigated the diurnal patterns of accelerometer-derived sedentary time, we only had information about the overall volume of specific sedentary behaviors. Future studies could use diaries to identify the times when specific sedentary behaviors take place. Lastly, older adults who were limited by their health to walk a couple of 100 meters were excluded from the current study. Therefore, our findings could not be generalized to older adults with such mobility impairment.

To conclude, our findings highlight the potential to tailor interventions aiming to reduce sedentary time to the needs of different sociodemographic subgroups of the older adult population. When addressing specific periods of the day, particular attention might be given to sedentary time during the evening. When aiming to reduce daytime sedentary time, older adults aged at least 75 years, and those widowed or non-married are important target groups. Older adults aged at least 75 years and men were at increased risk for higher levels of overall sedentary time and might, therefore, be considered as important target groups when developing interventions. Given that previous studies showed that high levels of television viewing time had more adverse health effects than other sedentary behaviors [24,25,52], the high volumes and increased risk for lower educated older adults observed in the current study, priority could be given to interventions targeting reductions in television viewing time among older adults with primary or secondary education. Interventions targeting reductions in sitting while driving a car or using the computer should devote special attention to older adults aged 65 to 74 years old, men and those with higher educational levels. Our findings provide starting points for the consideration of what might be the most appropriate programs and policies to address sedentary behaviors as a newly-understood health risk among older adults. Future research should examine which approaches are the most acceptable, feasible and effective to reduce sedentary time among older adults.
